# Supplementary material for: The intersection of food insecurity and child health: Implications for policy and practice in the Bronx
Source: PLoS One. 2025 Nov 20;20(11):e0335355. doi: 10.1371/journal.pone.0335355 (PMC12633916; doi:10.1371/journal.pone.0335355)
Supplement: S1 Table — (DOCX) [file pone.0335355.s001.docx]

**Supplemental Table 1: Missing Values for Outcome, Confounding Variables, and Food Insecurity Among Children in the Bronx, 2021**

|  | **#** | **%** |
| --- | --- | --- |
| **At risk of food insecurity** |  |  |
| Missing | 0 | 0 |
| **Age group** |  |  |
| Missing | 0 | 0 |
| **CHILD's Race/Ethnicity** |  |  |
| Missing | 0 | 0 |
| **Insurance Type** |  |  |
| Missing | 1 | 0.1 |
| **Housing Type** |  |  |
| Missing | 248 | 15.1 |
| **Parent Education Level** |  |  |
| Missing | 2 | 0.1 |
| **Parent Employment** |  |  |
| Missing | 4 | 0.2 |
| **Household Poverty Level** |  |  |
| Missing | 0 | 0 |
| **CHILD's Nativity** |  |  |
| Missing | 0 | 0 |
| **NYC born** |  |  |
| Missing | 1 | 0.1 |
| **Receipt of public assistance in past 12 months** |  |  |
| Missing | 6 | 0.36 |
| **Asthma** |  |  |
| Missing | 1 | 0.1 |
| **Obesity/Overweight** |  |  |
| Missing | 0 | 0 |
| **Depression** |  |  |
| Missing | 397 | 24.1 |
| **Anxiety** |  |  |
| Missing | 397 | 24.1 |
| Adjustment disorders |  |  |
| Missing | 399 | 24.2 |
| **Behavioral problems** |  |  |
| Missing | 398 | 24.2 |
| **Learning disorders** |  |  |
| Missing | 397 | 24.1 |
| **ADD/ADHD** |  |  |
| Missing | 398 | 24.2 |

Missing values are presented as unweighted counts and percentages.
