# Supplementary material for: The intersection of food insecurity and child health: Implications for policy and practice in the Bronx
Source: PLoS One. 2025 Nov 20;20(11):e0335355. doi: 10.1371/journal.pone.0335355 (PMC12633916; doi:10.1371/journal.pone.0335355)
Supplement: Attachment — Submitted filename: review_childhealth.docx [file pone.0335355.s002.docx]

**Overall**: This paper is well written, interesting, and touches on the important topic of child food insecurity risk and health. The authors however do not keep consistent the language about the outcome they measured in the survey. The survey includes the hunger vital sign questions – which produces estimates closer to marginal food security and not comparable to other measures of food insecurity. Therefore, the researchers main findings are incorrect. This paper could be published after this is addressed. I have included suggestions for future submissions.

**Suggested decision:**

This paper could be accepted after authors address the concerns.

**Summary:**

This paper uses survey data to investigate food insecurity -risk- in households with children in the Bronx. The summary of the prior literature is detailed, thorough and relevant. Researchers claim to find food insecurity in over half of households with children, a rate many times higher than other common estimates.

Heterogeneity in food insecurity risk by characteristics such as, race and income levels are also shown. This research additionally discusses that food insecure households in the Bronx areas face food access barriers as well as barriers to healthy eating. Authors state their contribution is that food insecurity rates in the Bronx are higher than the national average but they are not comparing food insecurity rates to food insecurity rates. They are comparing food insecurity rates to food insecurity risk, or marginal food insecurity.

**Detailed comments:**

- The primary exposure measure, risk for food insecurity, was assessed by the following questions: 1) “*Within the past 12 months, we worried whether our food would run out before we* *got money to buy more. Was this…”* and 2) *“Within the past 12 months, the food we bought* *didn’t last and we didn’t have money to get more. Was this…”* Response options for both questions were *“Often true”, “Sometimes true”, “Never true”, “Don’t know”, and “Missing”*. The latter two responses were recoded to missing. Participants who responded *“Often true”* or *“Sometimes true”* to either question were identified as being at risk of having food insecurity.

The main issue of the paper arises from the measures section. Authors do say they are measuring **Risk** of food insecurity but could elaborate more in this section and throughout the paper. For example, please explain to the reader why is outcome considered risk for food insecurity and not “food insecurity”. Authors should state that they do not use the full 18-item USDA-ERS measure, or the six item short form for measuring food insecurity.

Authors are not measuring food insecurity, what they are measuring is *marginal* food insecurity – commonly referred to as the Hunger Vital Sign.

Marginal food insecurity is a broader measure of food insecurity that captures households had problems at times, or anxiety about, accessing adequate food, but the quality, variety, and quantity of their food intake were not substantially reduced.

Authors could fix this mistake in a future draft by saying they are measuring marginal food insecurity, a less severe measure of food insecurity.

In some places the authors drop the word “risk” and just include “food insecurity”:

- For example, in the abstract authors do not always say food security **risk**– “Descriptive statistics and weighted multivariable logistic regression models were employed to examine the association between food insecurity and specified childhood health outcomes.”
- Additionally, in the results and discussion only food insecurity is used and not risk for food insecurity.
- **Controls only include a few characteristics:** Adjusted models included child age, child race/ethnicity, and household poverty level.
  - Other controls should be included in the model to control for confounding factors. Such as highest education in the household, parents employment status ( which you have in the data), parents marital status. All factors known to be associated with risk of food insecurity. Do you have any information on if the child or child’s family receives WIC, school meals or SNAP.
  - Insurance type should be included in the regressions.

Potentially helpful literature for the authors:

1) I know your survey was both online and telephone, but this article talks about why surveys may have higher rates of food insecurity.
   a. Can Internet Surveys Mimic Food Insecurity Rates Published by the US Government? Sunjin Ahn, Travis A. Smith, and F. Bailey Norwood* Applied Economic Perspectives and Policy (2020) volume 42, number 2, pp. 187–204. doi:10.1002/aepp.13002
