# Supplementary material for: The intersection of food insecurity and child health: Implications for policy and practice in the Bronx
Source: PLoS One. 2025 Nov 20;20(11):e0335355. doi: 10.1371/journal.pone.0335355 (PMC12633916; doi:10.1371/journal.pone.0335355)
Supplement: Attachment — Submitted filename: Revision of PLOS ONE article-LM-HH.docx [file pone.0335355.s003.docx]

**Revision of PLOS ONE article: The intersection of food insecurity and child health: implications for policy practice in the Bronx**

**Abstract:**

While the background provides a general overview, it lacks specificity in relation to the focus of the manuscript, particularly in terms of the physical and mental health outcomes addressed in the results. Providing more targeted context of these outcomes would help better frame the study’s relevance and align the abstract more closely with the study findings and discussion.

**Introduction**

The introduction is well-written and provides a strong foundation for the manuscript. However, it could be further strengthened by more clearly articulating why it is important to examine the health effects of food insecurity not only in adults but also in children. Highlighting this distinction would help contextualize the study’s contribution and underscore the urgency of addressing child food insecurity as a public health issue. Additionally, the flow of ideas could be enhanced by following a more structured progression. Consider starting with a clear definition of food insecurity, followed by its national prevalence in the US and how the COVID-19 pandemic affected this prevalence, then narrowing it down to NYC and its specific neighborhoods- particularly the Bronx, which is the central of the study. This funneling would provide a stronger rationale for the focus of the manuscript. Finally, then when discussing the health effects of food insecurity, it may help to categorize them into physical and mental health consequences, and discussing which consequences exactly are going to be studied in this manuscript (not just asthma in particular).

The introduction could also be enhanced by included somehow similar articles investigating the effect of food insecurity on mental health such as Itani et al., 2022 and Rahi et al., 2025.

- Lines 61-62: Unclear and does not align with the standard terminology. Consider rephrasing ‘nutritionally available food’ to ‘nutritionally adequate’, improving clarity and bettering aligning with public health terminology.

- Lines 65-68: Consider revising this sentence for clarity and flow of ideas.

-Line 78: Consider revising ‘medical effects’ to ‘medical outcomes’ or ‘health consequences.

- Line 82-102: The two paragraphs effectively highlight food insecurity among NYC residents especially the Bronx; however, consider revising the two paragraphs for improved clarity, conciseness, and flow of ideas to help emphasize the key messages better.

**Methodology**

-Line 144: Make sure that all reported numbers, particularly those referring to sample characteristics or results include both the count (n) and the corresponding percentage (%) consistently.

**Results**

Make sure that all reported numbers, particularly those referring to sample characteristics or results include both the count (n) and the corresponding percentage (%) consistently.

-Table 1: Categories row: consider revising the variables and rephrasing ‘households at risk of food insecurity vs households not at risk of food insecurity’ or ‘at risk households’ vs ‘low risk households’.

-Table 1: FPL has not been defined before. Include in table notes.

-Line 249: Adjustment disorders have not been mentioned before neither in the introduction nor in the methodology. This variable should be mentioned in the objectives of the study as well as the methodology.

**Discussion**

Lines 268-271: The two points mentioned here regarding the relationship between food insecurity and the health outcomes in children are important; however, they would be significantly strengthened by comparing the findings to those of other relevant studies. This helps better contextualize the study findings within the current body of research.

Lines 279-289: The proposed policy interventions are strong but could be further strengthened by elaborating on their implementation, feasibility, and expected impact. This would strengthen the practical relevance of the proposed policies and provide greater value to policymakers and practitioners.
