# Supplementary material for: The intersection of food insecurity and child health: Implications for policy and practice in the Bronx
Source: PLoS One. 2025 Nov 20;20(11):e0335355. doi: 10.1371/journal.pone.0335355 (PMC12633916; doi:10.1371/journal.pone.0335355)
Supplement: Attachment — Submitted filename: review_childhealth2.docx [file pone.0335355.s005.docx]

Thank you for the opportunity to review this article. The authors addressed the comments that I had. The article should be accepted.
