# Supplementary material for: The intersection of food insecurity and child health: Implications for policy and practice in the Bronx
Source: PLoS One. 2025 Nov 20;20(11):e0335355. doi: 10.1371/journal.pone.0335355 (PMC12633916; doi:10.1371/journal.pone.0335355)
Supplement: Attachment — Submitted filename: Response_to_Reviewers_auresp_2.docx [file pone.0335355.s007.docx]

October 7, 2025

Professor Andreas Beyerlein

Academic Editor, *PLOS ONE*

Manuscript ID: PONE-D-25-22823

Title: The intersection of food insecurity and child health: implications for policy and practice in the Bronx

Dear Reviewers and Editor,

We are grateful for the opportunity to resubmit our manuscript.

We appreciate the very thoughtful and thorough comments and suggestions made by the Associate Editor and reviewers. Please find attached the revised version of the manuscript with track changes as well as descriptions of all changes made to address each suggestion, as instructed in your earlier email. We are very pleased to report that we have been able to address all of the comments and suggestions, and we hope that you agree with us that the paper is substantially improved as a result. We hope that this revised manuscript is deemed deserving of publication in *PLOS ONE.*

Best regards,

Lily Lee

**Editor Comments:**

Response:

I do not understand the authors' response to my last issue: Under "Additional Information", they state: "All data is held in a public repository: <https://data.cityofnewyork.us/Health/2017-2021-NYC-KIDS-Survey/u7vp-i37z/about_data>". So how does this fit to their statement that the data are not publicly available? My main point was anyway that they would add their SAS analysis code to this repository and mention the URL in the Methods section. I see no data protection issues regarding this.

- We appreciate the reviewer’s comment. The link under “Additional Information” directs to the NYC Open Data portal which provides survey documentation and aggregate information, but not the full microdata. The microdata is not publicly available due to privacy and legal restrictions and can only be accessed through a data use agreement with the New York City Health Department. As our research team does not have access to upload files to the NYC Open Data portal, we have revised the manuscript to clarify this distinction and to state that the SAS analysis code will be made available from the corresponding author upon reasonable request.

Response:

Supplementary Table X seems to be missing (and should be declared as Supplementary Table 1).

- We apologize for forgetting to include the Supplementary Table in our original resubmission. It has not been included and has been declared as Supplementary Table 1.

Response:

I think it would be helpful for some readers if the authors could add a brief explanation how the weighting was conducted (e.g. with an example). Further, they should shortly discuss whether (and why possibly) some groups were underrepresented (e.g. older children) and whether this might have affected the results or not.

- Thank you for this helpful suggestion. We have added a concise description of how the NYC KIDS sample weights were derived and applied, as well as a brief discussion of potential underrepresentation in the data. Specifically, we note that the NYC KIDS sample weights account for unequal probabilities of selection, nonresponse, and frame coverage, and were post-stratified and calibrated to population totals. This ensures that estimates are representative of the NYC child population aged 1–13 years. We also added a statement acknowledging that some demographic subgroups (e.g., older children) may have been underrepresented due to differential nonresponse, though the post-stratification and raking procedures were designed to minimize resulting bias.

Response:
As a very minor point, the abbreviation "aOR" should not be used in the Abstract without explanation.

- The Abstract has been adjusted and the abbreviation “aOR” has been removed.
